# The Transcription Factor VvbHLH053 Regulates the Expression of Copper Homeostasis-Associated Genes *VvCTr5/6* and *VvFRO4* and Confers Root Development in Grapevine

**DOI:** 10.3390/ijms26010128

**Published:** 2024-12-26

**Authors:** Songqi Li, Xufei Li, Pengwei Jing, Min Li, Yadan Sun, Leilei Wang, Qiaofang Shi, Yihe Yu

**Affiliations:** 1 College of Horticulture and Plant Protection, Henan University of Science and Technology, Luoyang 471023, China; wnlsqsy@163.com (S.L.); lixufei8023@163.com (X.L.); jpw0612@163.com (P.J.); limin03301@163.com (M.L.); sunyadan@yeah.net (Y.S.); wqwq13027638395@163.com (L.W.); 9906384@haust.edu.cn (Q.S.); 2 Henan Provincial Engineering Research Center on Characteristic Berry Germplasm Innovation & Utilization, Luoyang 471023, China

**Keywords:** grapevine, chlormequat chloride (CCC), transcriptome sequencing, Cu homeostasis-associated genes

## Abstract

Chlormequat chloride (CCC) has been demonstrated to inhibit plant growth and strengthen seedlings. The present study demonstrated that the root growth of *Thompson seedless* grapevine seedlings was significantly enhanced by the application of CCC treatment. Nevertheless, the precise mechanism by which CCC regulates plant root growth remains to be elucidated. Consequently, an RNA-sequencing (RNA-Seq) analysis was conducted on grapevine roots subjected to CCC treatment and those undergoing natural growth. A total of 819 differentially expressed genes were identified. Subsequently, Gene Ontology (GO) functional enrichment and weighted gene co-expression network analysis (WGCNA) identified the Copper (Cu) homeostasis-associated genes, *VvCTr4/5/6/8* and *VvFRO4*, which play a pivotal role in mediating the effect of CCC. To further elucidate the transcription factor regulating these Cu homeostasis-associated genes, the key transcription factor VvbHLH053 was identified based on the PlantTFDB database, WGCNA results, and expression patterns under CCC treatment. Furthermore, multiple bHLH binding sites were identified on the promoters of *VvCTr4/5/6* and *VvFRO4*. The GUS activity analysis and dual-luciferase assay demonstrated that VvbHLH053 can directly regulate the expression of *VvCTr5/6* and *VvFRO4*. These findings reveal the feedback mechanism of grapevine root growth mediated by CCC and establish a direct functional relationship between CCC, VvbHLH053, and Cu homeostasis-associated genes that regulate root growth.

## 1. Introduction

The root is the foundation of plant growth and serves as an important organ for a number of essential functions, including support, absorption, synthesis, storage, secretion, drainage, reproduction, and regulation [1]. The growth status of the root directly affects the growth and development of the entire plant, and it plays a role that cannot be ignored. The root plays a pivotal role in the growth and development of perennial woody fruit trees, acting as the foundation and central axis of the tree’s overall structure [2]. The growth and development of plant roots are directly influenced by soil nutrients and soil moisture [3]. To ensure the normal growth and development of the root system, it is necessary to accumulate a sufficient amount of storage nutrients, photosynthetic products, and appropriate endogenous hormones. Furthermore, the nutrients and hormones must be properly coordinated in time and space, and a suitable external environment is required to establish a root group with a reasonable structure, suitable thickness ratio, and strong function [4,5].

The growth of the roots is primarily contingent upon the dimensions of the meristem and the length of the cells in the mature zone [6]. The maintenance of the root tip stem cell microenvironment, the division and differentiation of the meristem cells, and the elongation of cells in the elongation zone are all processes that regulate the size of the meristem and the final length of the cell, respectively. It can be concluded, therefore, that the factors affecting the aforementioned processes are all involved in regulating the elongation and growth of roots [7,8]. In *Arabidopsis*, the basic helix-loop-helix (bHLH) family transcription factor MYC2 has been demonstrated to directly bind to the promoter region of *PLETHORA (PLT)*, thereby inhibiting *PLT* expression and interfering with the maintenance of the root tip stem cell microenvironment. This ultimately results in the inhibition of root growth [9]. The soybean expansin gene *GmEXP1* is specifically expressed in roots, with higher levels of expression observed in the root tip elongation zone relative to the mature zone. The ectopic expression of *GmEXP1* in tobacco has been observed to result in accelerated root growth, which suggests that *GmEXP1* plays a role in regulating soybean root development [10]. Furthermore, heavy metal stress has been demonstrated to exert a direct influence on plant root growth [11]. It has been demonstrated that the accumulation of copper (Cu) in plant root cells can affect root development by modifying the proliferation rate of root meristem cells [12]. In *Arabidopsis thaliana*, the accumulation of Cu is dependent on the activity of Cu homeostasis-associated genes, including *COPPER TRANSPORTER (COPTs)*, *FERRIC REDUCTASE OXIDASE (FROs)*, *YELLOW STRIPELIKE* (YSLs), *ZINC-REGULATED TRANSPORTER IRON-REGULATED TRANSPORTER PROTEIN 2 (ZIPs)* and *COPPER CHAPERONE (CCH)* [13]. This suggests that these genes play a role in regulating root growth and development.

Plant growth regulators (PGRs) are artificially synthesized chemical substances that have similar effects to plant hormones and exert an effective regulatory effect on the growth and development of plant roots [14]. The molecular mechanism of the exogenous use of plant growth regulators affecting plant roots has also been extensively studied. The rice *DEEPER ROOTING 1 (DRO1)* gene encodes an auxin (IAA) responsive protein. Its expression is negatively regulated by auxin, which affects the elongation of cells at the root tip, leading to asymmetric root growth and an enhanced response to gravity [15]. In *Arabidopsis*, the promotion of SHY2 expression and the negative regulation of *PIN* expression by *AHK3/ARR1* and *AHK3/ARR12*, induced by cytokinin, results in a reduction in auxin accumulation and accelerating differentiation of root meristematic cell differentiation [6,16]. The gibberellin (GA) pathway has been demonstrated to promote root elongation and growth by targeting the degradation of DELLA proteins GAI and RGA, which act as the repressing components in the GA signaling pathway [17]. Chlormequat chloride (CCC) is a plant growth retardant that has been demonstrated to directly inhibit the biosynthesis of GA [18]. However, the molecular mechanism by which CCC regulates plant root growth remains to be elucidated.

The grapevine (*Vitis vinifera* L.) is one of the most significant cultivated fruit trees globally. It has been cultivated in China for over two millennia and is of immense economic value [19]. The grapevine is a deep-rooted fruit tree with a relatively developed root system. The root is fleshy and capable of storing a substantial quantity of nutrients, including a range of organic and inorganic components such as moisture, vitamins, starch, and sugar. In addition to the role of fixing plants, the function of the grapevine root system is primarily to absorb water and nutrients from the soil and to accumulate and store nutrients, thereby providing the material basis for the renewal and rejuvenation of the ground [20]. In the context of production, PGRs are frequently employed to regulate root growth in grapevine. The application of naphthalene acetic acid (NAA) has been demonstrated to effectively increase the number of grapevine roots and promote the growth of new roots [21]. In asexual cuttings, indole butyric acid (IBA) and indole acetic acid (IAA) are primarily employed to enhance rooting and elevate the reproductive rate [22].

The objective of this study was to investigate the effects of CCC on grapevine seedlings. The results demonstrated that CCC effectively promoted the growth and development of grapevine roots, increasing the number of roots. Furthermore, we elucidate the mechanism of VvbHLH053 in response to CCC treatment by regulating the expression of Cu homeostasis-associated genes *VvCTr5/6* and *VvFRO4.* The findings of our study offer new insights into the mechanism of CCC function, providing a theoretical basis and scientific guidance for its application in viticulture.

## 2. Results

### 2.1. Effect of CCC Treatment on the Growth of Grapevine

**Figure 1:**
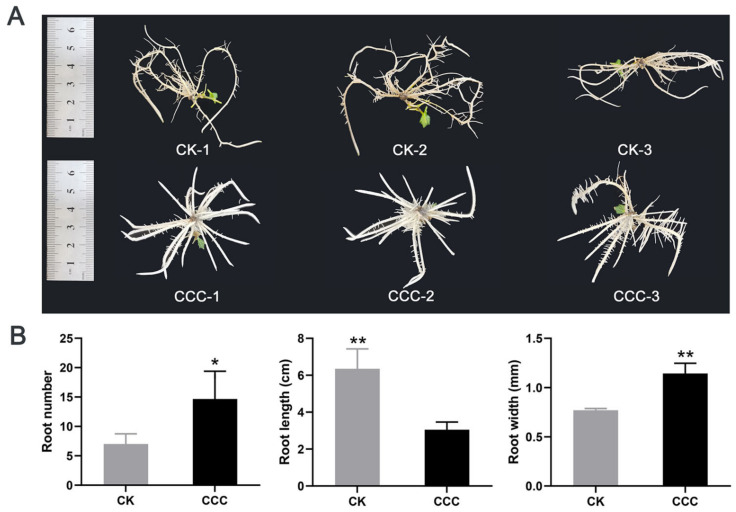
The effect of CCC on the root system of grapevine seedlings. (**A**) Alterations in the root phenotype of grapevine seedlings treated with CCC. The experiment was conducted with three biological replicates in both the treatment and control groups. (**B**) The statistical analysis of the root index encompasses the following parameters: root number, root length, and root width. The data presented are the mean ± standard deviation of three independent experiments, analyzed by Student’s *t*-test (* *p* < 0.05, ** *p* < 0.01).

In order to examine the impact of CCC on grapevine growth, grapevine stem segments were inoculated in a medium containing CCC. The application of CCC treatment resulted in notable morphological alterations to the roots of grapevine cuttings (Figure 1A). Further observation and measurement of the root system through phenotypic analysis revealed that, in response to the CCC treatment, root elongation growth was inhibited, while the number of lateral roots increased, and the roots themselves became more robust (Figure 1B). Furthermore, the roots of the CCC-treated groups exhibited a phenotype characterized by enhanced brightness and an increase in root hairs. The observed phenotypic alterations suggest that CCC facilitates the transition of the root system to a state conducive to plant growth. This implies that CCC can enhance the root development of grapevine seedlings.

### 2.2. Qualitative Evaluation of RNA-Seq Data

In order to gain insight into the molecular mechanism by which CCC promotes grapevine root development, transcriptome sequencing was conducted on the seedling roots of the treatment group and the control group. The sequencing results demonstrated that the total number of clean reads was 65,671,956 and 66,050,067 in the control and CCC treatment groups, respectively. The alignment rate of these high-quality reads with the genome of the ‘Pinot noir’ grapevine was 89.49% to 91.19%. Each sample produced more than 6G clean bases, the proportion of Q20 exceeding 97% and a proportion of Q30 exceeding 92%. The GC content was greater than 45% (Supplementary Table S1). The sequencing data were of excellent quality and could meet the requirements of subsequent analysis.

**Figure 2:**
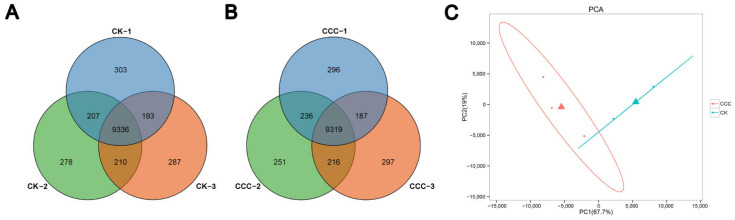
The number of expressed genes in the various root samples and the results of PCA. (**A**) The number of expressed genes in three biological replicates of root samples from a normal growing grapevine. (**B**) The number of expressed genes in three biological replicates of root samples treated with CCC. (**C**) PCA biplot of control and CCC-treated samples.

A total of 42,973 genes were identified in the transcriptome data set, of which 10,039, 10,031, and 10,026 genes were expressed in the control group designated CK-1, CK-2, and CK-3, respectively, and 10,038, 10,022, and 10,019 genes were expressed in the treatment group designated CCC-1, CCC-2, and CCC-3, respectively. As illustrated in the Venn diagram, 9336 genes were co-expressed in all control groups (Figure 2A), and 9319 genes were co-expressed in all CCC-treated groups (Figure 2B). The aforementioned genes were subsequently subjected to further analysis. To assess the consistency of the three samples from the same treatment, principal component analysis (PCA) was conducted. The first and second principal components collectively explained 67.7% and 19% of the total variance, respectively (Figure 2C). The samples from both the control and treatment groups exhibited excellent reproducibility and distinct separation from one another. The results demonstrated differential gene expression between the control and the treated groups.

### 2.3. Identification and Functional Enrichment of Differentially Expressed Genes (DEGs)

**Figure 3:**
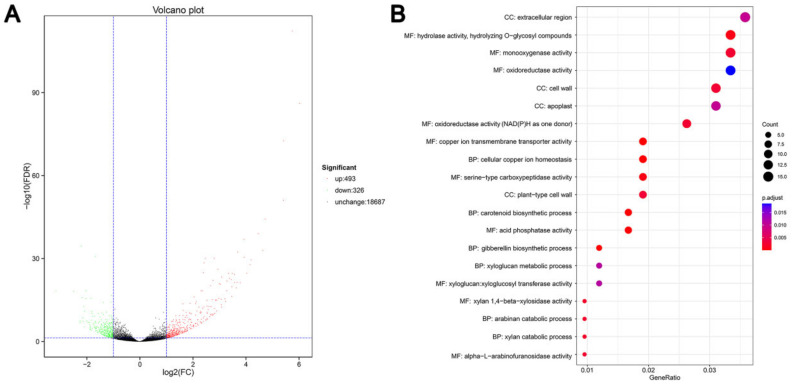
The volcano plot and GO function of DEGs. (**A**) Volcano plot of DEGs in the table; each dot represents a gene. The plot depicts the expression levels of genes, with red dots representing those that are up-regulated, green dots representing those that are down-regulated, and black dots representing those that are not differentially expressed. (**B**) The top 20 most significantly enriched GO functional terms for the up-regulated DEGs. The figure depicts a visual representation of the GO function, wherein each circle represents a specific function. The color of the circle is indicative of the FDR value, and the size of the circle is proportional to the number of those enriched within that function. The three categories are as follows: biological processes (BP), cellular components (CC), and molecular functions (MF).

A differential expression analysis was conducted to identify statistically significant DEGs between the control and CCC-treated groups. The criteria employed to determine statistically significant DEGs were an absolute fold-change value of ≥2 and a false discovery rate (FDR) of <0.05. The volcano map indicated that the expression of 819 genes in grapevine roots underwent a significant alteration following CCC treatment, with 493 genes exhibiting increased expression and 326 genes displaying decreased expression (Figure 3A). The number of genes that were up-regulated was significantly higher than that of genes that were down-regulated in both groups of samples. Consequently, further analysis was focused on the up-regulated genes.

To gain insight into the biological functions of these DEGs, we conducted a Gene Ontology (GO) enrichment analysis. A total of 133 up-regulated genes were found to be enriched in 37 GO functional annotations, comprising 18 biological processes, 13 cellular components, and 6 molecular functions. The DEGs involved in biological processes were primarily associated with cellular copper ion homeostasis, cell wall biogenesis, and the catabolic process of cell wall polysaccharides. Additionally, they were involved in the biosynthetic and metabolic processes of hormones. With regard to cellular components, the distribution of the DEGs was predominantly observed in the cell wall, the apoplast, and the cell cortex. The molecular function of the DEGs was predominantly associated with binding and catalytic activity (Figure 3B). These findings indicate that genes involved in copper ion transport, cell wall formation, and hormone metabolism may play a significant role in the promotion of grapevine root development by CCC.

### 2.4. Co-Expression Network of Genes Related to Root Development

**Figure 4:**
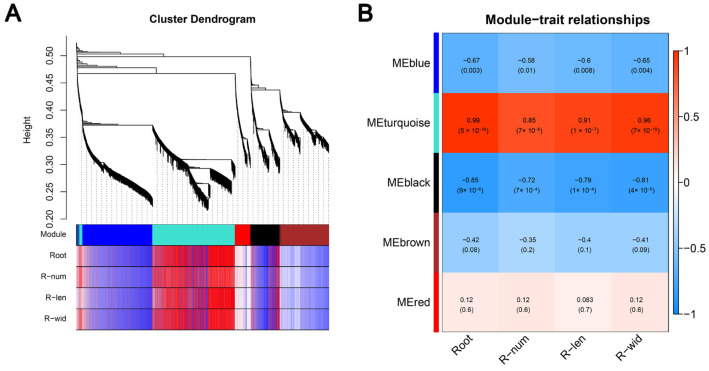
The WGCNA was employed to identify genes associated with plant root development. (**A**) Hierarchical cluster tree displaying the co-expression of gene modules. Each branch of the dendrogram represented a cluster of interconnected genes, which were grouped together into a module. The modules colored accordingly are displayed in the lower panel. (**B**) The correlation between gene expression modules and plant roots and their respective indexes (root number, root length, and root width) across the entire co-expression gene network. The left panel depicts five modules, each represented by a distinct color. The right-hand panel presents a color scale for the features between modules, with values ranging from −1 to 1. The correlation value and *p*-value are displayed for each module.

In order to identify the gene regulatory networks related to plant root development, a weighted gene co-expression network analysis (WGCNA) was performed, and the correlation between the expression patterns and changes in root number, root length, and root width was studied. The pairwise correlation analysis system based on gene expression clustered five co-expressed WGCNA modules, designated blue, turquoise, black, brown, and red, were identified (Figure 4A). The results demonstrated that the related indexes of root development exhibited the highest positive correlation with the gene expression of the turquoise module (r ≥ 0.85, *p* ≤ 7 × 10^−6^) and the highest negative correlation with the gene expression of the black module (r ≤ −0.72, *p* ≤ 7 × 10^−4^). The turquoise module, which exhibited the most positive correlation with plant root development, was subjected to further analysis, encompassing the 2828 genes identified therein.

### 2.5. Cu Homeostasis-Associated Genes Involved in Grapevine Root Growth Induced by CCC

**Figure 5:**
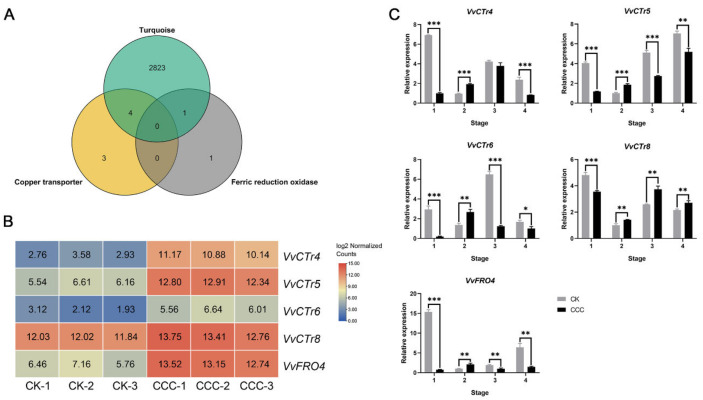
Identification and expression analysis of candidate genes involved in the root growth of grapevine induced by CCC. (**A**) The number of genes within the GO pathway that are associated with copper homeostasis in the turquoise module. (**B**) Heatmap analysis of *VvCTr4/5/6/8* and *VvFRO4* expression in grapevine roots after CCC treatment. The data used the log2 values of the DESeq2 normalized gene counts. (**C**) The expression patterns of *VvCTr4/5/6/8* and *VvFRO4* in grapevine roots at four stages under normal growth and CCC treatment. The error bars represent ± standard deviation (SD) (n = 3). Significant differences in values were determined by Student’s *t*-test (* *p* < 0.05, ** *p* < 0.01, and *** *p* < 0.001).

The results of the GO enrichment and WGCNA analyses revealed the presence of multiple genes involved in maintaining Cu homeostasis in plants within the turquoise module. This suggests that these genes may play a role in the process of grapevine root growth induced by CCC (Figure 5A). The genes associated with copper homeostasis include four members of the COPT/CTr family: *VvCTr4* (Vitvi06g01729), *VvCTr5* (Vitvi06g01730), *VvCTr6* (Vitvi06g00559), and *VvCTr8* (Vitvi04g01813). Additionally, there is one FRO gene, *VvFRO4* (Vitvi16g01091). The heatmap demonstrated the expression of these genes in the transcriptome (Figure 5B). Furthermore, the expression of these genes in the different stages of grapevine root development induced by CCC was investigated by using Quantitative Real-Time Polymerase Chain Reaction (qRT-PCR) analysis. The results demonstrated that during the second period, the expression pattern of these genes was consistent with that observed in the transcriptome under the corresponding treatment. It is noteworthy that the expression pattern of these genes in the initial stage was contrary to that observed in the transcriptome and that the expression of these genes was markedly repressed by CCC treatment. In the third or fourth stage, only *VvCTr8* exhibited continued up-regulation following CCC treatment, while the expression of the remaining genes was significantly diminished in comparison to the control group (Figure 5B).

### 2.6. VvbHLH053 Directly Regulate the Expression of VvCTr5/6 and VvFRO4

**Figure 6:**
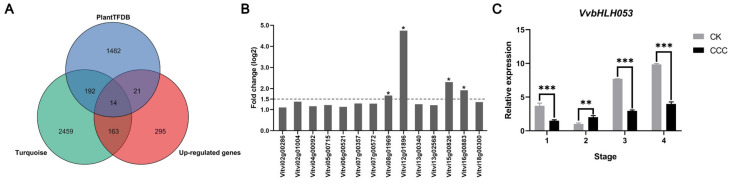
Identification of potential TFs of Cu homeostasis-associated genes. (**A**) The number of TFs exhibiting analogous expression patterns with regard to Cu homeostasis-associated genes within the turquoise module. (**B**) The expression of 14 TFs in the transcriptome was quantified using the log2 values of the ratio between the CCC treatment and the control group. TFs with the log2 values of the fold change exceeding 1.5 were deemed to be of particular significance and are indicated with an asterisk. (**C**) The expression patterns of *VvbHLH053* in different periods under CCC treatment. The error bars represent ± standard deviation (SD) (n = 3). Significant differences in values were determined by Student’s *t*-test (* *p* < 0.05, ** *p* < 0.01, and *** *p* < 0.001).

In order to identify the potential transcription factors (TFs) of Cu homeostasis-associated genes associated with grapevine root growth induced by CCC, an analysis of the up-regulated genes in the turquoise module was conducted. This analysis was performed using the grapevine transcription factor information available in the PlantTFDB 4.0 database, resulting in the identification of 14 TFs (Figure 6A). Of the transcription factors identified, four exhibited significant differential expression following CCC treatment. Of these, the most prominent was identified as Vitvi12g01896, a member of the grapevine bHLH transcription factor family and designated *VvbHLH053* (Figure 6B). The results of the qRT-PCR demonstrated that the expression pattern of genes associated with copper homeostasis exhibited a similar trend to that of *VvbHLH053* throughout the process of grapevine root growth induced by CCC. In the second stage, CCC induced the expression of *VvbHLH053*, while in the first, third, and fourth stages, the expression of *VvbHLH053* in the control group was significantly higher than that in the CCC group (Figure 6C).

**Figure 7:**
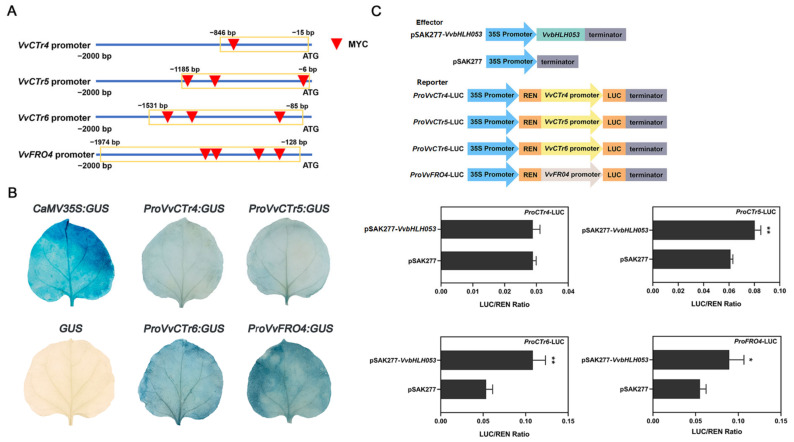
VvbHLH053 promotes the transcription of Cu homeostasis-associated genes by binding to the MYC present elements in their respective promoters. (**A**) Predicted MYC elements in the promoters of *VvCTr4/5/6* and *VvFRO4*. The blue lines represent the promoter sequences of *VvCTr4/5/6* and *VvFRO4*. The red inverted triangles indicate the bHLH binding sites, designated as MYC. The golden rectangles represent the promoter fragments that were used for cloning purposes. (**B**) Analysis of GUS activities of the *VvCTr4/5/6* and *VvFRO4* promoters. The *GUS* gene lacking a promoter was employed as the negative control, while the *GUS* gene under the control of the CaMV 35S promoter served as the positive control. (**C**) Dual-luciferase assay. The upper part of the figure illustrates the schematic of the effector and reporter constructs, while the lower part depicts the impact of *VvbHLH053* overexpression on the transcriptional output of *VvCTr4/5/6* and *VvFRO4* promoters. The empty pSAK277 vector was employed as the control. The data are presented as mean ± SD (n = 3 replicates). Following the application of the Student’s *t*-test, the presence of statistically significant differences is indicated by the use of asterisks. * *p* < 0.05, ** *p* < 0.01.

Further investigation of the promoters of five Cu homeostasis-associated genes revealed the presence of multiple bHLH binding sites (MYC, CANNTG) in the *VvCTr4/5/6* and *VvFRO4* promoters, indicating that they may be transcriptionally regulated by bHLH TFs (Figure 7A). Conversely, no such site was identified in the promoter of *VvCTr8*, suggesting that bHLH TFs are not involved in regulating the transcription of *VvCTr8*. To ascertain whether the promoters of these genes were active, the MYC elements were cloned from the promoter sequences, and the GUS activity of the promoters was analyzed. The results demonstrated that the promoters of *VvCTr4/5/6* and *VvFRO4* exhibited promoter activity and were capable of initiating gene expression (Figure 7B). Subsequently, we employed the dual-luciferase reporter system to ascertain whether VvbHLH053 could directly regulate the four Cu homeostasis-associated genes. The results demonstrated that the overexpression of VvbHLH053 markedly activated the promoter activities of *VvCTr5/6* and *VvFRO4*, whereas it had no discernible impact on the transcriptional activity of *VvCTr4* (Figure 7C). These findings indicate that VvbHLH053 directly binds to the *VvCTr5/6* and *VvFRO4* promoters, thereby inducing their expression.

## 3. Discussion

The plant growth-inhibiting hormone CCC has a number of physiological functions, including the control of vegetative growth, the promotion of dwarfing, the enhancement of tillering, the stimulation of reproductive growth, and the increase of fruit setting. Additionally, it has been demonstrated to enhance the ability of plants to withstand drought, cold, and saline conditions [23]. The application of CCC resulted in the development of stronger roots and an increase in the number of roots in grapevine seedlings. However, root elongation was inhibited by CCC, which may be attributed to the fact that CCC, acting as an antagonist of GA, impairs the function of GA [24].

Prior research has demonstrated that CCC exerts control over plant growth by influencing the absorption of essential elements by plants. In rice, CCC treatment has been demonstrated to influence the accumulation of sodium (Na) and potassium (K), regulate aboveground growth, and enhance yield [25]. In Avena, treatment with CCC affected the calcium (Ca) and magnesium contents in yellowing leaves [26]. Transcriptome analysis revealed that multiple gene family members associated with copper homeostasis exhibited differential expression in response to CCC treatment, including COPT, FRO, YSL, ZIP, and CCH gene families (Supplementary Table S2). This indicates that CCC facilitates grapevine root development, presumably by regulating the accumulation of Cu. Cu is a vital trace element for plant growth and development, playing a pivotal role in numerous biological processes within plants, including photosynthesis, respiration, elimination of reactive oxygen species, cell wall synthesis, and hormone signal transduction [27]. As a vital organ for the absorption of nutrients, the growth of the root system is directly influenced by Cu. An excessive accumulation of Cu will result in toxic effects on plants. A high concentration of Cu in grapevine roots has been observed to result in a number of adverse effects, including root growth inhibition, root darkening, a reduction in root hair numbers, and root elongation [28]. A low concentration of Cu stress has a beneficial effect on plants, promoting root growth. Recent studies have demonstrated that low concentrations of Cu can stimulate the growth rate of the root system of grapevine [29].

The absorption of Cu by plants is dependent on the participation of COPT/Ctr and FRO proteins in the root system [30,31]. The expression of *COPT/Ctr* and *FRO* genes exerts a direct influence on the absorption and accumulation of Cu in plants, which in turn affects root growth. In *Arabidopsis thaliana*, the overexpression of COPT1 resulted in elevated copper accumulation and the inhibition of root growth. Conversely, the accumulation of Cu was reduced, and root growth was enhanced in the *copt1* mutant [32,33]. Additionally, Cu accumulation was found to be diminished in the *fro4* mutant, with concomitant effects on root growth [34]. The grapevine *VvCTr1* gene exhibits rapid responsiveness to exogenous Cu, participates in Cu absorption and transport, and plays a crucial role in maintaining root growth of *copt5*-deficient *Arabidopsis* seedlings [35]. In this study, the expression of *VvCTr4/5/6/8* and *VvFRO4* was found to be inhibited by CCC treatment during the early rooting stage of grapevine seedlings and to gradually increase with growth. It is hypothesized that CCC inhibits the absorption and accumulation of Cu by inhibiting the expression of *VvCTrs* and *VvFRO4* in grapevine, thus directly affecting the growth of the root system. The expression pattern of *VvCTr8* differs from that of the other *VvCTr* genes, which may be associated with their distinct functions. In *Arabidopsis*, the *COPT1/2/6* genes are responsible for absorbing external Cu, while the *COPT3/5* genes are involved in the Cu transport process within the plant [30]. Furthermore, plants regulate Cu homeostasis by modulating Cu metabolism and reducing Cu consumption by inhibiting the expression of numerous Cu-containing proteins, Cu/Zn superoxide dismutases (CSD1 and CSD2), LACCASEs (LACs), and plastocyanin (ARPN). This enables the maintenance of optimal plant growth when copper supply is limited [36]. The results of transcriptome analysis indicated that the transcription of multiple CSD, LAC, and ARPN family members was significantly inhibited (Supplementary Table S2). These findings align with our initial hypothesis that the Cu content in grape roots was initially low during the early stage of CCC treatment. Furthermore, the increased transcription levels of COPT and FRO family members in the later stage of CCC treatment may also be associated with the low accumulation of Cu.

**Figure 8:**
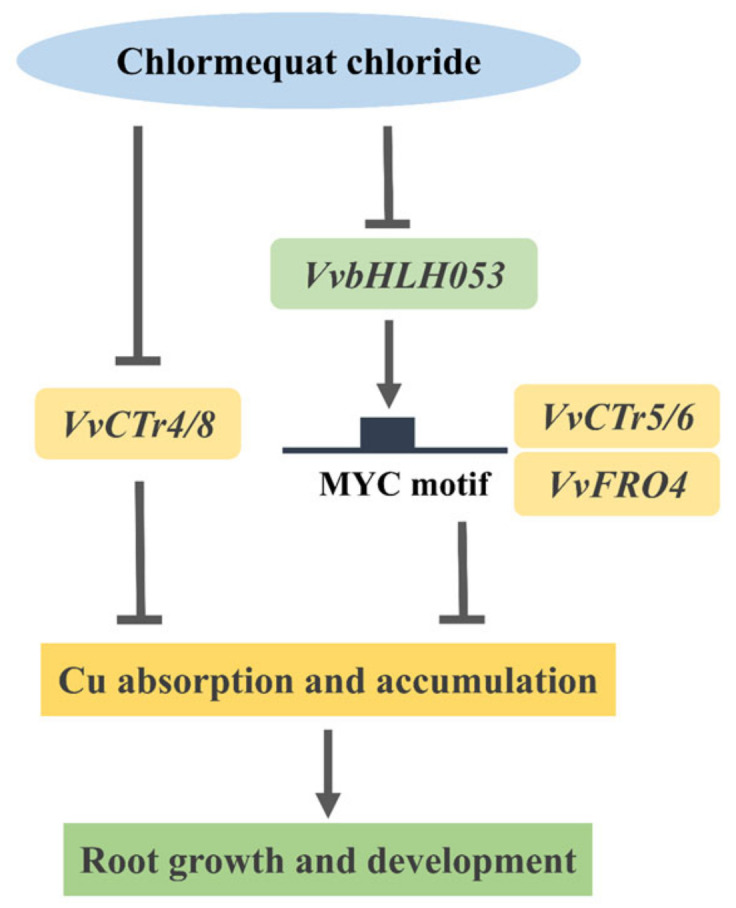
Model of *VvbHLH053* and Cu homeostasis-associated genes function in grapevine under CCC treatment. The solid lines represent direct regulatory relationships that have been validated by experimental data.

The bHLH protein family constitutes a substantial subset of transcription factors within plant transcription networks. These factors are involved in a number of crucial biological processes, including the regulation of cell elongation and the development of fruits, flowers, and roots. Additionally, they play a role in the transduction of plant hormones [37]. Prior research has demonstrated that Arabidopsis bHLH family transcription factors are also instrumental in the regulation of metal homeostasis. CITF1 (bHLH160), a transcription factor involved in the response to Cu deficiency, has been demonstrated to directly regulate the expression of genes associated with copper homeostasis, specifically *COPT2* and *FRO4/5*. This regulation affects the absorption of Cu by roots and promotes root growth [38]. The present study revealed that the grapevine bHLH family member *VvbHLH053* exhibited differential expression following CCC treatment. It has been reported that *VvbHLH053* is the homologous gene of *CITF1* [39]. It may, therefore, be hypothesized that analogous transcriptional regulation patterns may also be present in the grapevine. Further investigation revealed that the expression pattern of *VvbHLH053* was similar to that of the Cu homeostasis-associated genes. Furthermore, the presence of multiple bHLH binding sites in the promoters of *VvCTr4/5/6* and *VvFRO4* indicates the potential for regulation VvbHLH053. Finally, the ability of VvbHLH053 to act as a positive regulator, directly influencing the expression of *VvCTr5/6* and *VvFRO4*, was confirmed through a dual-luciferase assay. It was hypothesized that CCC inhibits the expression of *VvbHLH053*, which in turn inhibits the transcription of Cu homeostasis-associated genes *VvCTr5/6* and *VvFRO4* in the early growth stage of grapevine seedlings. This results in a reduction in the absorption and accumulation of Cu, thereby promoting the growth and development of the roots (Figure 8).

## 4. Materials and Methods

### 4.1. Plant Material and Treatment

The study employed the grapevine variety of *Thompson seedless*. The stem segments, measuring 3 cm in length, derived from grapevine seedlings, were employed as explants for cuttings. These stem segments were inoculated in a solid medium comprising half-strength Murashige and Skoog salts (Macklin, Shanghai, China) as control groups. Furthermore, the treatment groups were added with CCC (200 mg/L) [18]. The regenerated plants were cultivated in a chamber at 25 °C with a 16 h day/8 h night cycle. Four samples were obtained at various stages of the explants’ development into complete plants. The initial sampling was conducted on the 16th day of explant growth on the medium, at which point the plants were in the early stages of root development. Subsequent samplings were performed at 7-day intervals (days 23, 30, and 37). In each period, three plants exhibiting similar growth characteristics were selected from the treatment and control groups, respectively, for sampling. Phenotypic analysis was conducted in the fourth stage to more clearly illustrate the influence of CCC treatment on grape roots. RNA-seq sequencing was conducted on the samples from the second stage to ascertain the genetic alterations that underpin the early effects of CCC treatment on the growth of grape roots. The roots of the collected plants are promptly enveloped in aluminum foil and frozen in liquid nitrogen, then stored at −80 °C for subsequent analysis.

### 4.2. RNA Sequencing Analysis

The plant root samples were preserved in freezing tubes and subsequently dispatched to Beijing Biomarker Biotechnology Co., Ltd. (Beijing, China) for transcriptome sequencing. The non-strand-specific libraries were constructed in Illumina Novaseq 6000 (Illumina, Hayward, CA, USA), and paired-end sequencing was performed. Subsequently, the Illumina sequencing reads for each sample were aligned to the grapevine reference genome sequence (http://ftp.ensemblgenomes.org/pub/plants/release-32/fasta/vitis_vinifera/ (accessed on 19 February 2021)). The HISAT2 tool (version 2.2.1) can be employed to efficiently align RNA-Seq reads, while StringTie is a suitable choice for assembling reads derived from the alignments. The raw sequences were submitted to the NCBI database under the BioProject accession number PRJNA788660.

### 4.3. Analysis of DEGs

The DEseq2 R (version 4.1.2) package was employed for differential analysis, and genes exhibiting a fold change of ≥2 and a false discovery rate (FDR) of <0.05 were identified as DEGs between the two samples. In order to reduce transcriptional noise, the expression of the gene was considered. If the count value ≥1 in at least one sample, then the gene was included in further analysis. The DESeq2 normalized gene counts were employed as normalized gene counts. The TBtools software (version 2.056) was employed to generate a visual representation of the gene expression heatmap based on the log2 values of the DESeq2 normalized gene counts. Transcription factor annotation was performed using the PlantTFDB database (http://planttfdb.gao-lab.org/ (accessed on 26 November 2022)).

### 4.4. GO Enrichment Analysis

The GO enrichment analysis of DEGs was conducted using the clusterProfiler R (version 4.1.2) package. In order to ascertain whether the GO terms in question were significantly enriched, a comparison was made with the GO database (http://www.geneontology.org/ (accessed on 29 October 2023)) using an FDR-adjusted *p*-value of <0.05.

### 4.5. WGCNA and Visualization of Gene Networks

The WGCNA was conducted using the WGCNA package in R (version 4.1.2) to identify genes in modules that were associated with root growth traits. The modules were obtained through the automatic network building function, which was set to the default parameters. To ensure the continuity of the WGCNA dataset, thresholding was set to 0.1, allowing for the identification of candidate central genes with positive correlation.

### 4.6. RNA Extraction and qRT-PCR

The total RNA was extracted from root samples using an RNAprep Pure Plant Kit (TIANGEN, Beijing, China) in accordance with the manufacturer’s instructions. First-strand cDNA was synthesized using the HiScript III-RT SuperMix (Vazyme, Nanjing, China) for qPCR. qRT-PCR was performed using the TransStart Top Green qPCR SuperMix kit (Transgen, Beijing, China). The *VvUbiquitin 1* gene was employed as the internal control [40]. The relative expression levels of the genes were determined by the 2^−ΔΔCt^ method. The primer sequences for the genes were designed by Primer Premier 5.0 software and are listed in Supplementary Table S3.

### 4.7. GUS Activity Analysis

The promoter sequences located upstream of the ATG codon for *VvCTr4/5/6* and *VvFRO4* were cloned by PCR from *Thompson seedless*. The amplified promoters were then fused to the HindIII/EcoRI sites of the 0390-35S-GUS vector by homologous recombination, employing a seamless cloning kit (Beyotime, Shanghai, China). The *CaMV35S:GUS* construct was employed as a positive control, while *GUS* was utilized as a negative control. The *CaMV35S:GUS*, *GUS*, *ProVvCTr4/5/6:GUS*, and *ProVvFRO4:GUS* recombinant plasmids were transformed into *Agrobacterium tumefaciens* strain GV3101 (Weidi, Shanghai, China) using the freeze–thaw method. The experimental material, comprising four-week-old *Nicotiana benthamiana* leaves, was infected with *Agrobacterium tumefaciens* permeation solution using the vacuum permeation method. Following a period of 16 h of illumination and 8 h of darkness, the leaves were subjected to staining with a GUS kit (Huayueyang, Beijing, China) in accordance with the instructions provided. The leaves were decolorized with ethanol until the green pigmentation was completely absent in order to observe GUS activity. The primers utilized for these promoters are provided in Supplementary Table S3.

### 4.8. Dual-Luciferase (Dual-LUC) Assay

The promoters of *VvCTr4/5/6* and *VvFRO4*, which contain HindIII/BamHI restriction sites, were ligated to the pGreen II 0800-Luc vector. The coding sequence (CDS) of *VvbHLH053* was amplified from the cDNA of grapevine roots and subsequently fused to the EcoRI site of the vector pSAK277. The recombinant plasmids of *ProVvCTr4/5/6-LUC* and *ProVvFRO4-LUC* were employed as reporters, while the plasmid pSAK277-*VvbHLH053*, which was under the control of the cauliflower mosaic virus (CaMV) 35S promoter, was used as the effector. The reporter vectors were transformed into *Agrobacterium tumefaciens* GV3101, which also harbored the pSoup plasmid. The vector of the effector was transformed into GV3101. The *Agrobacterium* cells containing the reporter and effector constructs were co-injected into the abaxial surfaces of *Nicotiana benthamiana* leaves. Subsequently, the tobacco was cultivated in the dark for one day, followed by normal light for two days to detect the activity of luminescence. The Dual-Luciferase Reporter^®^ Assay System (Promega, Madison, WI, USA) was employed to ascertain the Firefly luciferase (LUC) and Renilla luciferase (REN) fluorescent values in accordance with the instructions provided. The experiment was conducted in triplicate. The primers employed are detailed in Supplementary Table S3.

## 5. Conclusions

The findings of this study indicate that CCC has the potential to stimulate root development and thickening in grapevine seedlings while simultaneously inhibiting elongation growth. Transcriptome sequencing revealed that the CCC treatment influenced the expression of genes associated with copper homeostasis, including *VvCTr4/5/6/8* and *VvFRO4*. These genes regulate copper absorption and accumulation and are essential for root growth and development. Furthermore, the transcription factor *VvbHLH053* has been observed to directly bind to the promoter regions of *VvCTr5/6* and *VvFRO4*, thereby regulating their expression. The present study proposed a response model of Cu homeostasis-associated genes to CCC treatment (Figure 8), which provides a foundation for further investigation into the molecular mechanisms by which CCC affects root growth and development.

## Data Availability

https://www.ncbi.nlm.nih.gov/bioproject/PRJNA788660/ (accessed on 15 October 2024).

## Supplementary Materials

The following supporting information can be downloaded at https://www.mdpi.com/article/10.3390/ijms26010128/s1.
